# Osteochondroma of rib

**DOI:** 10.11604/pamj.2022.42.59.35217

**Published:** 2022-05-23

**Authors:** Prateek Upadhyay

**Affiliations:** 1 Department of Orthopaedics, Jawaharlal Nehru Medical College, Datta Meghe Institute of Medical Sciences (Deemed to be University), Wardha, Maharashtra, India

**Keywords:** Ostechondroma, rib, vetebrae

## Image in medicine

Osteochondroma is also known as exostosis and is a benign bone tumour. It commonly presents in the first 3 decades of life with a male preponderance. The most common site for the development of these tumours is the metaphysis of long bones, with almost 30% of cases originating from the distal metaphysis of the femur. Origin from flat bones like ilium and scapula is rare, while origin from vertebrae and ribs is unheard of. A 21-year-old female presented with a history of swelling over the left upper back since 2 years, pain in the left upper back and shoulder with tingling since 4 months. On examination, a 3 cm by 3 cm, hard, immobile mass was palpable in the left paravertebral region of the 4^th^ intercostal space. X-ray and computed tomography (CT) scan of the thorax revealed two irregular lesions arising from the neck of the 5^th^ rib posteriorly and a Fine Needle Aspiration Cytology (FNAC) revealed the lesion to be of chondroid matrix origin, consisting of normal chondrocytes. Through a posterior approach, two pedunculated tumours of size 3.5 x 2 x 1 cm and 2 x 2 x 1 cm, along with the costovertebral junction of the 5^th^ rib were resected. Histopathological examination confirmed the mass to be an osteochondroma of the rib. On 6 months follow up, the patient is stable, has no pain or tingling and there is no evidence of recurrence of the tumour.

**Figure 1:**
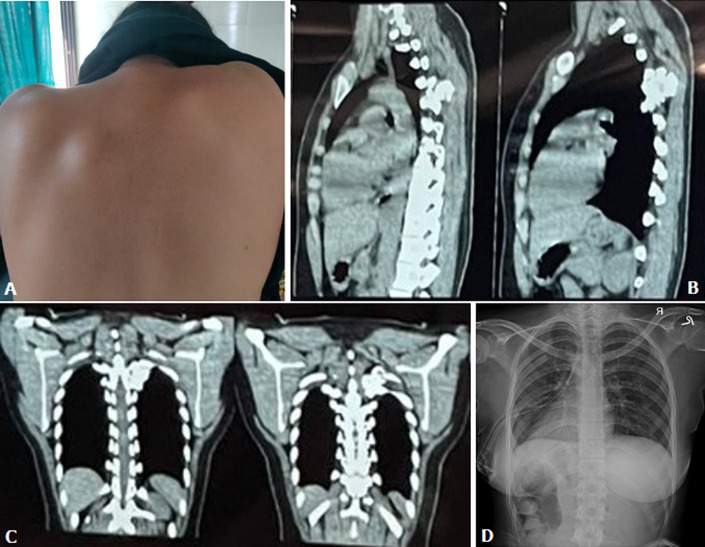
A) clinical picture of the swelling in the left 4^th^ intercostal region; B) sagittal section of CT thorax showing the irregular mass between the 4^th^ and 5^th^ rib; C) coronal section of CT thorax showing the irregular mass between the 4^th^ and 5^th^ rib; D) post-operative X-ray with resected mass and the costovertebral junction of the 5^th^ rib

